# A Promising Tool in Serological Diagnosis: Current Research Progress of Antigenic Epitopes in Infectious Diseases

**DOI:** 10.3390/pathogens11101095

**Published:** 2022-09-25

**Authors:** Jiahuan Zhou, Jiayi Chen, Yunchi Peng, Yafeng Xie, Yongjian Xiao

**Affiliations:** Department of Clinical Laboratory, The Second Affiliated Hospital, Hengyang Medical School, University of South China, Hengyang 421001, China

**Keywords:** infectious diseases, B cell epitope, epitope prediction, diagnosis, biomarker

## Abstract

Infectious diseases, caused by various pathogens in the clinic, threaten the safety of human life, are harmful to physical and mental health, and also increase economic burdens on society. Infections are a complex mechanism of interaction between pathogenic microorganisms and their host. Identification of the causative agent of the infection is vital for the diagnosis and treatment of diseases. Etiological laboratory diagnostic tests are therefore essential to identify pathogens. However, due to its rapidity and automation, the serological diagnostic test is among the methods of great significance for the diagnosis of infections with the basis of detecting antigens or antibodies in body fluids clinically. Epitopes, as a special chemical group that determines the specificity of antigens and the basic unit of inducing immune responses, play an important role in the study of immune responses. Identifying the epitopes of a pathogen may contribute to the development of a vaccine to prevent disease, the diagnosis of the corresponding disease, and the determination of different stages of the disease. Moreover, both the preparation of neutralizing antibodies based on useful epitopes and the assembly of several associated epitopes can be used in the treatment of disease. Epitopes can be divided into B cell epitopes and T cell epitopes; B cell epitopes stimulate the body to produce antibodies and are therefore commonly used as targets for the design of serological diagnostic experiments. Meanwhile, epitopes can fall into two possible categories: linear and conformational. This article reviews the role of B cell epitopes in the clinical diagnosis of infectious diseases.

## 1. Introduction

Although the mass production of vaccines and widespread use of antibiotics have decreased the burden of infectious diseases in recent decades, infectious diseases remain a major cause of death at present [1]. In particular, under conditions such as growing population density and changes in urbanization and climate, some emerging infectious diseases have sprung up continually. In addition, several notorious pathogens have increased in the spread, and other pathogens have even evolved a multidrug-resistant strain, causing increasing difficulty in terms of treatment [2]. Given that there are no characteristic or typical clinical symptoms for most infectious diseases, laboratory tests are therefore essential to identify pathogens. They typically consist of etiological laboratory examinations and general laboratory examinations. General laboratory examinations, such as C reactive protein, procalcitonin, and complete blood count, are nonspecific screening programs for infectious diseases. It is evident that etiological examinations are particularly crucial to identify the cause of a disease. In terms of etiological laboratory examinations, isolation and identification of pathogens are the best processes to carry out for the diagnosis of infectious diseases; however, it is time-consuming to isolate, culture, and identify pathogens from suspicious specimens. Furthermore, positive rates are low because the sensitivity of the culture is easily affected by the quantity and quality of the specimen material [3]. Microscopy is another important detection method that observes pathogens directly, but the results are vulnerable to uncertain factors such as the technician’s experience, morphological variability, and a load of pathogens in the samples. Additionally, pathogen-related components, including antigens, antibodies, toxins, nucleic acids, and metabolites, are also listed as etiological laboratory examinations to help characterize pathogens as soon as possible. In recent years, molecular diagnostic technologies, as new tools of laboratory diagnosis with high sensitivity and specificity, have been continuously implemented in clinical laboratories. Currently, polymerase chain reaction (PCR) is widely used in clinical diagnosis, but it is easily contaminated and its operating conditions are strict. Moreover, the serological test is also a suitable option that can generate a rapid result clinically. However, many pathogens, especially similar pathogens, have cross-reaction in serological reactions, which makes the diagnosis of the infection difficult. Furthermore, seroassay cannot clearly distinguish the course of the disease. It has been the goal that many researchers have been pursuing to improve the specificity and sensitivity of serological diagnostic tests. Fortunately, due to the emergence of epitopes, an attempt to design an epitope-based serological test has been proposed. As for clinical diagnosis, the selection and combination of epitopes for the design of diagnostic reagents can improve the sensitivity and specificity of methodology and eliminate cross-reaction. Additionally, the combination of epitopes has the potential to distinguish different courses of disease. Epitopes refer to the specific determinants that play an important role in antigenicity. Another term opposite to the concept of epitopes is called paratopes, referring to the part of the antibody that binds to the antigen-binding sites in the antigen–antibody complex [4,5]. Since the concept of epitopes has been put forward, a great number of researchers have been interested in exploring the binding sites on antigens. Hence, a variety of experimental methods were adopted to determine the structure of epitopes, but they were found to be unsatisfactory due to being time-consuming, laborious, and costly. In recent years, coupled with the development of bioinformatics technology and related theoretical knowledge, a vast number of epitope prediction methods have been thriving.

Epitopes data have accumulated rapidly in the past decade. It is of high importance to analyze epitopes for numerous medical, immunological, and biological applications including infectious diseases’ prevention, diagnostics, and treatment [6,7].

This paper reviews the research progress of B cell epitope recognition and prediction, and summarizes the application of B cell epitopes in the diagnosis of infectious diseases.

## 2. Definition and Classification

Epitopes (antigenic determinants) are a chemical group with special structure and immune activity on the surface of antigenic molecules, and represent an immunoreactive region on antigenic molecules. They are able to stimulate the body to produce antibodies or sensitized lymphocytes and can be recognized by the generated antibodies or sensitized lymphocytes, which typically account for only 5–7 amino acid residues, with no more than 20 amino acid residues at most. Based on their spatial structure, epitopes have been divided into continuous and discontinuous epitopes. Discontinuous epitopes, also known as conformational epitopes (CEs), are composed of a group of residues that are not contiguous in sequences, and are brought together by the folding of a protein to its native structure in space. Continuous epitopes, also known as linear epitopes (LEs), consist of a stretch of residues that are sequentially connected in the polypeptide sequence [4,8]. Alternatively, epitopes can also be classified into B cell epitopes and T cell epitopes in the light of their targeting receptor [9]. In brief, T cell epitopes, recognized by T cell receptors (TCRs), are continuous epitopes, submitted through the major histocompatibility complex (MHC) molecule. MHCI molecules present endogenous antigens, whose epitopes are recognized (identified) by CD8+ TCRs, whereas MHCII molecules present exogenous antigens, recognized by CD4+ T cells [10]. Unlike T cell epitopes, the majority of B cell epitopes, binding to B cell receptors (BCRs), immunoglobulins (Igs), or antibodies, are discontinuous epitopes (continuous epitopes only account for 10%) [7,11,12]. The underdevelopment of current technology has led to the real spatial structure and function of CEs not being completely simulated; thus, LEs remain the primary focus.

## 3. Epitope Mapping and Prediction

**Figure 1:**
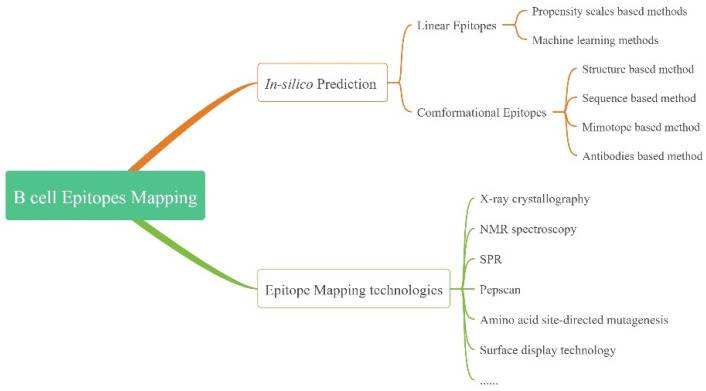
Schematic logic of the third section in this paper.

The identification of B cell epitopes holds enormous potential for disease prevention, diagnosis and treatment. This ultimately falls into two types: epitope mapping in experiments and in silico epitope prediction (Figure 1).

### 3.1. Prediction

As bioinformatics technology and multifarious databases have continued to mature and develop, approaches and software for epitope analysis have been massively boosted (Table 1). 

#### 3.1.1. LEs

At present, the B cell epitope is the hotspot in epitopes research, as the binding of it with BCRs can stimulate B cells to produce antibodies. Continuous studies have focused on prediction, and methods for computational prediction have also made great progress. Primary sequence-based information on antigens was used for linear epitope prediction in early in silico methods. Initially, the prediction approaches for epitopes were based on amino acid propensity scales depicting the physicochemical features of B cell antigenic determinants. The most classic and earliest prediction parameter is the hydrophilic parameter proposed by Hopp and Woods in the 1980s based on the assumption that hydrophilic amino acids, considered as potentially antigenic, are generally located on the surface of antigen molecules, while hydrophobic amino acids are embedded inside [13]. Over time, other amino acid propensity scales for B cell epitope prediction were revealed successionally, such as flexibility, polarity, solvent accessibility, secondary structure, surface accessibility, β-turn propensity, etc. [7,10,11]. The accuracy of a single parameter did not perform as well as expected and applications were basically of no use in practice. Thus, several propensity scales have been combined to predict B cell LEs and to design software such as PEOPLE and BcePred [14,15]. The accuracy of methods based on multiple propensity scales is certainly higher than based on one; however, one experiment that combined scales to evaluate prediction performance found that the specificity and sensitivity scores for optimized scales set were only marginally better than the randomized ones [16].

With the emergence of a variety of machine learning (ML) algorithms, researchers have implemented them in the field of epitopes prediction. Among ML-based methods, the fundamental processes are to first collect a comprehensive epitope dataset; then, extract the features of the antigen sequences; and finally, obtain a trained model by using ML algorithms to train on the collected sample dataset. Thus, a new epoch for epitopes prediction has been established. For example, one prediction method, named BepiPred [17], was first proposed and published through the combination of classical parameters with the hidden Markov model (HMM), an ML algorithm. The receiver operating characteristic (ROC) curve score of BepiPred is 0.671 and its specificity is 80% through testing. The notion that the accuracy of BepiPred was superior to other methods at that time was reported by Larsen’s team [17]. Several months later, a server, ABCpred [18], based on artificial neural network (ANN), was also published. Non-redundant epitopes and non-epitope peptides from the Bcipep and SwissProt databases were extracted as training sets; the predictive sensitivity reached up to about 67% and the specificity reached 65%. Then, BepiPred upgraded to BepiPred-2.0 [19], and is a well-known predictor so far. As for ML-based methods, to date, numerous researchers have devoted themselves to developing different ML-based methods to improve the accuracy of B cell LE prediction. These approaches have significantly accelerated progress on the application of B cell epitopes.

#### 3.1.2. CEs

Since it is generally accepted that B cell CEs account for the majority of B cell epitopes, many studies have been focused on B cell CEs prediction in recent years. However, CEs are discontinuous, making prediction difficult. Existing methods for conformational epitope prediction can be broadly classified into four categories: structure-based methods, sequence-based methods, mimotope-based methods and antibody-based methods. CEP software was the first server, the algorithm of which utilizes the accessibility of residues and spatial distance cutoff of solvent-exposed residues, to predict B cell CEs based on the 3D structure of antigens [20]. DiscoTope, another CEs prediction software proposed in 2006, is trained on a dataset of discontinuous epitopes determined by X-ray crystallography [21]. With 95% specificity, DiscoTope was able to detect 15.5% of the residues at discontinuous epitopes [21]. Furthermore, the software ElliPro [22], SEPPA [23], etc., are all methods based on antigenic structure. To evaluate the performance of prediction tools, an independent dataset was created by collecting experimentally confirmed discontinuous epitopes tools. SEPPA resulted in the best performance with an average area under the curve (AUC) value of 0.62 and sensitivity of 0.49, while the AUCs of all other methods mentioned are around 0.5, which indicates that the prediction performance of these methods is not satisfactory [7]. Simultaneously, some studies have also indicated that these methods do not increase the accuracy of prediction performance [24].

Similar to LEs prediction, the antigen sequence-based method has also been introduced into the prediction of CEs; due to the expensive and time-consuming determination process, the inadequate number of currently available structures has limited the development of structure-based methods [7,25]. The principle of sequence-based methods is the use of the feature vector or matrix obtained by scoring each amino acid in the input protein chain to predict the antigenic epitope residues. However, a drawback of these programs is that they do not group predicted epitope residues into corresponding epitopes by approach [11,26]; therefore, further research is needed due to its unsatisfactory predictive performance.

In addition, a combinatorial approach, also known as the mimotope-based method, has been proposed that requires both the 3D structure of the antigen and the antibody affinity peptide sequence as input [7]. The principle of the method is to screen the antibody affinity peptides, defined as mimotopes, by screening the random peptide library using a monoclonal antibody (mAb). Mimotope and genuine epitopes generally have high sequence similarity and high affinity to paratopes of mAbs. Thus, genuine CEs have a higher chance of being obtained by mapping the mimotopes back to the source antigen [24]. MimoPro [27] and MIMOX [28], among others, are prediction tools developed through the theory of mimotopes. 

Moreover, there is another kind of method that takes antibody information into account. One of the earlier methods was proposed by Soga et al., in which the antibody-specific epitope propensity (ASEP) index was proposed and exploited to narrow down candidate epitope residues for individual antibodies. The advantages of it are that the paratopes are easy to predict and the ASEP index can eliminate part of the base residues [29]. EpiPred [30] and PEASE [31], etc., are antibody-based methods. The current evaluation of existing antibody-based epitope prediction methods is inadequate because the performance of each method is reported for different datasets [32].

### 3.2. Epitope Mapping Technologies

A strategy to identify epitopes in experiments would be equally welcomed in studies of immunology. Several common methods used for the identification of B cell epitopes are introduced below.

X-ray crystallography: Due to it being able to provide an unequivocal, atomic resolution picture of the antibody–antigen interaction by means of analyzing the atomic structure of the antigen–antibody complex, this method is believed to be the gold standard to identify the B cell epitopes. However, the apparatus is expensive and the operation process is much more complicated because of the need for high-purity antigens and antibodies and the final eutectic formation [37].

Nuclear magnetic resonance spectroscopy (NMR): In a high-intensity magnetic field, the nuclei of different molecules can absorb electromagnetic radiation of appropriate frequency and produce different resonance spectra. The nuclear magnetic resonance signal of the antigen itself is different from that of the antigen–mAb complex. Recording and dealing with this spectrum can accurately determine the amino terminal sequence of antigenic epitopes. This method can quickly and accurately determine the amino acid motif of the B cell epitope. However, this technique for B cell epitopes’ identification in the conventional laboratory is limited by its complicated operation process and expensive equipment [38,39,40]. In addition, the method is restricted to small proteins and peptides.

Surface plasmon resonance (SPR): Incident light illuminates the prism side of the surface, in total internal reflection conditions, which generates an evanescent wave [41]. Evanescent waves can propagate into the metal film and surface plasmons are excited in the metal surface layer. SPR occurs when the wave vector of the evanescent wave is equal to that of the surface plasmons [42,43]. Due to its advantages of high sensitivity, no requirements for additional biomarkers and its ability to be dynamically tracked, SPR has been used in a variety of biosensors [44]. Epitopes are identified by measuring the relevant parameters of the binding of antigenic epitopes and antibodies [43]. However, it also has certain disadvantages such as low sensitivity for small molecule detection, limitations on multiple analyses, and high environmental requirements for the SPR sensor [45].

Peptide scanning (Pepscan): According to the amino acid sequence of the specific protein molecule, consecutive overlapping peptides are synthesized; then, these peptides react with the corresponding specific antibodies one by one to determine the epitopes through enzyme-linked immunosorbent assay (ELISA), Western blotting (WB), protein chip or other immunological techniques. This technique requires a clear primary structure of the antigen, and the detection result is a linear epitope of the antigen [46,47]. Unfortunately, Pepscan is unable to provide complete epitope information.

Amino acid site-directed mutagenesis: A mutation takes place in a particular gene by using correlating techniques to produce the desired mutant protein; then, the antigenic epitopes of it can be identified by analyzing the binding of antigens and antibodies. In this method, both CEs and LEs can be studied [48,49]. However, it is an approach that is very laborious and time-consuming: it takes a fairly long time to obtain the protein for each mutation and there is a limit to the number of mutations that can be added at one time [50].

Surface display technology: Based on the distinct expression system, the technology has almost been developed for display technologies of phage, bacteria and yeast surfaces, which have similar methodology. The gene of the target fragment is introduced into the genome of the expression system, and then, the desired protein is expressed on the surface of the expression vector. Finally, the epitopes of the protein are depicted and identified using associated techniques [51,52]. Phage display technology is one of the classic and widely used technologies whose hallmark characteristic is that both the phenotype (peptide displayed on the phage surface) and genotype (coding sequence inside the phage) are present in the same system [53]. Moreover, it is able to produce very large libraries of peptides to achieve high-throughput screening. Meanwhile, its high stability and easy operation are also advantages of its widespread application [50,53,54].

In addition to the methods mentioned above, there are many other technologies that are applied to identify epitopes, such as mass spectrometry, chemical cleavage or biological enzymatic hydrolysis, flow cytometry peptide microarray, etc. Among methods, some can be used to identify both B cell epitopes and T cell epitopes. Moreover, the accuracy of epitopes identification has clearly improved when in combination with two or more methods. For instance, the combined application of surface display technology and flow cytometry technology can shorten the test cycle and reduce the cost; an intuitive and high-throughput transformation of the process can be achieved through flow cytometry technology. Each of the above methods has its own advantages and disadvantages; researchers can choose the most suitable method according to their own preferences and experimental conditions (Table 2).

## 4. Applications in Diagnosis

The diagnosis of infectious diseases has long been a difficult problem in clinics. Particularly over the last decade, outbreaks caused by pathogens such as severe acute respiratory syndrome coronavirus 2 (SARS-CoV-2), Ebolavirus (EBOV), and influenza virus have increased in frequency, emphasizing the urgency for the rapid development of diagnostics [2]. Although certain pathogens can be diagnosed by means of traditional culture methods, most pathogens are still difficult to identify because of factors such as the existence of fastidious bacteria (such as *Mycobacterium tuberculosis*) and the use of antibiotics before culture. Fortunately, serological diagnosis can alleviate the above problems to a large extent. This is due to its reaction not being affected by the morphology and load of bacteria, as well as the fact that generally IgM reflects recent infections and IgG reflects past infections. However, the sensitivity and specificity of serological laboratory diagnosis are limited by certain factors such as cross-reaction and variation in pathogens; thus, the emergence of epitope-based serological experiments is helpful to improve the accuracy of tests. Currently, with the development of techniques for screening and identification of epitopes, the complexity and cost of the determination of epitopes have been partially reduced. Accordingly, epitopes have received more and more attention in pathogen diagnosis in recent years.

### 4.1. Diagnosis in Bacterial Infection

Bacterial infection has always accounted for a relatively large proportion of infectious diseases. In recent years, the importance of epitopes in bacterial diagnosis has been proven in many studies. *M. tuberculosis* can invade every organ of the body, with pulmonary tuberculosis (TB) being the most common. The period of isolation and culture is long, and the sensitivity of smear microscopy is low. At the end of the last century, one research study compared the genome of pathogenic *M. tuberculosis, M. bovis,* and Bacille Calmette–Guérin vaccine strains by whole-genome DNA microarray, and found 16 genomic regions of difference (RD) [55]. Some of these regions have been studied in greater detail by later generations. It is well established that uses of the 10-kDa culture filtrate protein (CFP10) and 6-kDa early secreted target antigen (ESAT-6) proteins for the detection of TB. The CFP10 and ESAT-6 play important roles in mycobacterial virulence and pathogenesis through a 1:1 complex formation, thus one publication reported that they have found one novel B cell epitope of the complex protein, exhibiting about the same effect as the complex protein, and may be useful in the serodiagnosis of TB [56]. Sarcoidosis is a disease with symptoms similar to smear-negative pulmonary TB, but the management of them is entirely different. To rapidly differentiate them in serology, the combination of peptides from RD1 and RD2 was constructed, yielding 83.3% sensitivity for smear-positive, 62.5% for smear-negative, and only 4.16% for sarcoidosis [57]. The latest article reported a novel polypeptide molecule consisting of T cell epitopes and B cell epitopes predicted online for several proteins associated with latent TB infection and predicted online for the molecule’s associated parameters, suggesting its promise as a molecule for the prevention and or diagnosis of latent TB disease [58]. Overall, several proteins in these regions show strong immunogenicity and are candidates for the diagnosis of TB, but then the sensitivity as well as the specificity of both these proteins and epitopes, still needs to be improved.

In addition, *Staphylococcus aureus* is an opportunistic pathogen capable of causing a wide variety of infections, ranging from skin infections to severe invasive infections and even necrotizing pneumonia and sepsis [59,60,61]. Routine culture remains an accurate way to detect *S. aureus*, but it is time-consuming. Particularly for blood-borne infections caused by *S. aureus*, early administration of antibiotics has been reported to have a positive influence on survival. Therefore, rapid and inexpensive detection of *S. aureus* infection is a necessity in clinics. One of the directions is convenient serological tests based on epitopes. As early as the last century, experiments have shown that ELISA methods for quantitative detection of toxins have been developed by selecting epitopes of *S. aureus* enterotoxins to synthesize peptides that can be used to produce mAbs [62]. Besides, it was found that the pentasacyl bridge of peptidoglycan of *S. aureus* has high antigenic specificity. The covalently coupling peptide haptens, therefore, were synthesized by several scholars in view of the two specific representative polypeptide epitopes, PSau5 and PSau7. Then, a competitive ELISA was successfully established with high specificity and detectability to simplify the detection process and shorten the detection time [63].

Leptospirosis is an acute infectious disease with a natural focus. Importantly, leptospirosis urgently needs to be diagnosed as early as possible, as symptoms of it are ambiguous, easily leading to misdiagnosis [64]. As a result, researchers are working to develop more specific tools [65,66,67]. *Leptospira* is double membrane bacterium whose outer membrane proteins (OMPs) are potential targets for bacterial-host interactions and are therefore the focus of current epitope studies [65,66,67,68,69]. For instance, given that HAP1/LipL32 is an outer membrane protein present in pathogenic bacteria but not in putrefactive bacteria, and that the immunogenicity and diagnostic ability of this recombinant protein have been previously demonstrated, a specific amino acid sequence of this protein was selected to improve its initial diagnostic performance. A peptide antigen consisting of 26 amino acids was synthesized and was found to be of high value in early diagnosis, especially for IgG with a specificity of 100%; the antipeptide reaction was earlier than the detection of agglutinating antibodies in the microscopic agglutination test (MAT) [69]. Some other epitopes of outer membrane proteins, such as OmpL1, LipL21, LigA, have also been investigated, among which two epitopes of LigA have been used in combination to diagnose patients with acute leptospirosis with sensitivities and specificities up to 97.9% and 99.1 %, respectively [65]. In addition, it is worth noting that two studies both produced mAbs against different epitopes for disease diagnosis. The first predicted and synthesized LigA (LK90)-based B-cell-specific epitopes—LK90 (543) and LK90 (1110)—and then the corresponding mAbs were produced—P1B1 and P4W2—used for detecting urine samples through mAb-based dot blot ELISA. The specificity and sensitivity of the test were found to be in the range of 93–96% and 77–89%, respectively. It has been confirmed as a promising method for diagnosis due to its advantages of simplicity and high specificity [67]. Meanwhile, the other study designed antibodies for N-terminal immunogenic domains of LipL21, and also evaluated its specificity and sensitivity. Compared with the former, it is almost the same in specificity, but its sensitivity is not as good [66]. Besides, there are also some pieces of the literature on bacterial epitopes found here, especially the epitopes of two pathogens, *brucella* and *Borrelia burgdorferi*, which have been studied carefully in recent years and have yielded good diagnostic results, which have been put into the table below (Table 3). With the in-depth study of epitopes in diagnosis, it is reasonable to suggest that it is of innovative significance to the existing serological diagnosis. 

### 4.2. Diagnosis in Viral Infection

Viruses, a type of non-cellular organism, can cause serious diseases in humans. Many viruses (for example, SARS-CoV-2) are highly infectious, causing a serious economic burden on society. Thus, early diagnosis and effective treatment are the main measures to prevent the spread and aggravation of the virus. Though it is universally accepted that the isolation and culture of viruses are the standard methods for virus identification, the limitation that viruses are difficult to culture in vitro hinders widespread application in clinics. Additionally, PCR is ever-increasingly applied in diagnosing and determining the replication status of viral infections by detecting viral nucleic acid. However, it is expensive due to its instruments and is easily contaminated. In fact, the serological test has become one of the main methods for the fast diagnosis of viral infection in laboratories because of its rapidity and simplicity. False-positives caused by cross-reaction between viruses and the insensitivity of some reactions are still weaknesses of serological methodology; thus, the in-depth study of epitopes is a suitable choice to solve these problems.

#### 4.2.1. SARS-CoV-2

Regarding viruses, the first that comes to mind is SARS-CoV-2. The SARS-CoV-2 pandemic rages on in some parts of the world, constituting a global public health emergency [87]. It is crucial to develop a rapid diagnostics tool to control the spread of Coronavirus disease 2019 (COVID-19). As a consequence, a large number of studies have been carried out successively on epitopes of SARS-CoV-2. The neo-coronavirus particle consists of an RNA gene chain, four structural proteins (spike (S) protein, small envelope (E) glycoprotein and membrane (M) glycoprotein, nucleocapsid (N) protein) and several auxiliary open reading frame (ORF) proteins. Among them is the S protein [88,89,90,91,92,93], which binds to angiotensin-converting enzyme 2 on the surface of human cells and is involved in the fusion and replication of viral and human cells; and the N protein [91,93,94], which is relatively conserved and against which the body can produce high levels of antibodies early in infection. These have both become major foci in the development of diagnostic epitopes for new coronaviruses. As computational tools are convenient and enable the development of new detection methods to be accelerated, a number of studies have used them to predict the epitopes of proteins that diagnostic performance has been initially characterized as well [90,92,93,95]. For example, the synthetic peptides from S protein, predicted by online servers, were developed into a magnetic chemiluminescence enzyme immunoassay for the detection of SARS-CoV-2 antibodies, greatly enhancing the diagnostic accuracy of COVID-19 [90]. Another report stated that two B-cell linear epitopes of S protein, P104 and P82, were identified by screening peptide libraries overlapping S protein with convalescent serum. Among them, P104 was a newly identified epitope with an advantage for the identification of patients with asymptomatic infection, and the combined peptides, S14P5+S21P2+P104, exhibited a positive response rate of 86.7% for asymptomatic infections [89]. Moreover, one research team not only excavated the epitopes with high specificity and sensitivity on S and N proteins, but also found that there were some epitopes in which the response amplitude of IgG to them was closely related to the severity of the disease [96]. In addition to the two proteins mentioned above, other proteins have been studied, such as ORF8, which is not a structural protein but is highly immunogenic, so the prediction of its epitope is equally useful for the development of diagnostic reagents [97]. 

#### 4.2.2. Epstein–Barr Virus (EBV)

EBV, known as human herpesvirus 4, can lead to many diseases, including infectious mononucleosis, lymphoma, nasopharyngeal carcinoma (NPC), etc. It is a virus commonly susceptible to almost all populations and can cause serious physical damage to patients [98,99,100]. As serological testing is available and important for diagnosing EBV infection, as well as usually being targeted by the viral capsid antigen, EBV nuclear antigen and early antigen, the introduction of epitopes will greatly facilitate the development of serological diagnosis. Therefore, four different peptide mimotopes of these antigens—F1, A3, gp125, and A2—were selected for more accurate detection of EBV. The results showed that these peptide mimotopes could clearly distinguish EBV-positive and EBV-negative sera. Furthermore, through different combinations of peptide mimotopes, the sensitivity and specificity of the reaction were as high as 97.5% and 100%, respectively. This shows the potential of peptide mimotopes as a substitute for existing antigens used in the serological diagnosis of EBV infection [101]. Additionally, it is well known that EBV is closely associated with certain tumors, especially NPC. Studies have shown that EBV latent membrane protein 2 (LMP2) plays an important role in the pathogenesis of EBV-associated tumors. Therefore, many scientists have focused on the epitope of EBV LMP2, expecting to design a promising screening and diagnostic tool for NPC [98,102,103]. Among them, a novel B cell multi-epitope peptide fusion protein (EBV-LMP2-3B) has been constructed, which consists of three linear B cell epitopes of EBV LMP2. The sensitivity and specificity of this epitope in the diagnosis of NPC were as high as 91.91% and 93.14%, respectively [98].

#### 4.2.3. Dengue Virus (DENV)

DENV, an arbovirus of high concern, causes a range of symptoms, from mild fever to severe hemorrhagic fever, and even shock. Its non-specific clinical symptoms, especially the early mild fever, prevent timely and accurate diagnosis of dengue infection, eventually resulting in the progression of infection [104]. Nevertheless, the current problem of serology testing—a routine test for identifying DENV infection—is the cross-reaction between flaviviruses, so targeted efforts are needed to find more specific epitopes as diagnostic antigens in order to reduce the occurrence of cross-reaction and improve the specificity of the method. The structural E protein of DENV is one of the main antigenic targets of humoral immune response. Non-structural protein 1 (NS1) is secreted by infected cells in the early stage of infection and there are commercial reagents to detect the NS1 of DENV for the diagnosis of early infection [105]. Therefore, most of the existing diagnostic epitopes to date are aimed at E protein [106,107,108,109,110,111] and NS1 protein [108,112,113]. Dengue fever virus can be divided into four serotypes according to its antigenicity. Depending on the assay needs, researchers need to find different target epitopes. For example, in order to be able to correctly diagnose a certain serotype of DENV, 14 B cell epitopes of DENV type 1 bounded to the specific MAb of DENV-1—15F3-1—have been shortlisted. Then, P14M, the only synthetic peptide of DENV-1, has presented the ability to bind to 15F3-1 specifically and inhibit the binding of phage particles to 15F3-1. Thus, the peptide was used to diagnose DENV-1-infected patients as an epitope-based diagnostic tool, with 95% sensitivity and 100% specificity [112]. Furthermore, in order to quickly diagnose all dengue virus serotypes and avoid cross-reactions between the crude DENV extracts and other flaviviruses, a tetra-epitope peptide that selected one to four serotypes from E protein within the DENV was expressed by a plant expression system, with a sensitivity of 71.7% and specificity of 100% [106]. Moreover, another important result observed was the capturing of a conserved peptide that has the latent capacity to recognize all four DENV serotypes and distinguish them from Zika virus-positive serotype [114]. Thus, it is clear that the epitope has extraordinary prospects in the diagnosis of DENV.

#### 4.2.4. Hepatitis Virus 

Viral hepatitis, caused by a group of viruses that primarily attack the liver, is a major global public health problem. Hepatitis B virus (HBV) and hepatitis C virus (HCV) are also major contributors of leading to chronic infections and cirrhosis. The serological test has long been a mainstay of viral hepatitis testing, an important method for the clinical judgment of infection status is to detect the antigen or antibody. Even if available commercial kits have been widely applied in medical settings, it is still the continuous pursuit of the laboratory to improve the accuracy of detection. The value of epitopes has gradually been uncovered in the diagnosis of hepatitis virus. For the detection of anti-HBc antibodies, a study selected HBcAg epitopes to design a multi-epitope recombinant protein, being stable and inexpensive, whose detection ability was validated to be consistent with commercial antigens [115]. Additionally, hepatitis B e antigen (HBeAg) can be used as a marker for clinical detection of chronic hepatitis B. However, due to its high amino acid sequence homology with the hepatitis B core antigen (HBcAg), most of the existing anti-HBe antibodies cross-react with HBcAg, which greatly interferes with the accurate detection of HBeAg. The difference between HBeAg and HBcAg has become a point of concern. The SKLCLG (aa -10 to -5) motif on the N-terminal residues of HBeAg was found to be absent in HBcAg, so a novel mAb 16D9 against this epitope was prepared. Meanwhile, in conjunction with another mAb—14A7 mAb against the HBeAg C-terminus (STLPETTVVRRRGR, aa141 to 154)—one team has developed a method to detect HBeAg, which effectively eliminates the previously mentioned cross-reaction and improves the accuracy of e antigen detection [116]. The strategy of multi-epitope recombinant proteins is also applicable to detect HCV [117,118], and the recombinant protein designed back in 2006 achieved 100% sensitivity and 99.8% specificity for the detection of anti-hepatitis C antibodies. Due to the high mutation rate of HCV, the difficulty of detection and genotyping has increased. Therefore, four B-cell epitope peptides were constructed, including sequences targeting three genotypes of HCV, based on the analysis and epitopes prediction of conserved sequences in the E2 region of HCV, providing a new idea of thinking about serotyping for HCV [119]. To overcome the difficulty in diagnosing occult HCV infection, the enzyme immunoassay using a core antigen dominant epitope-derived peptide to detect IgG anti-HCV core was found to have a total of 40.7% positive rate in occult HCV-infected patients, indicating that epitope polypeptide is assistant to identifying occult HCV infection [120]. Although the action of epitopes for the genotyping of hepatitis viruses and the detection of occult infection remains to be explored, epitopes perform relatively well in the routine diagnosis of hepatitis virus infection.

#### 4.2.5. Ebolavirus (EBOV)

Filoviruses cause rare but deadly viral hemorrhagic fevers—EBOV and Marburgvirus (MBV) are two formidable agents among them. Even though the detection of RNA is more accurate, the two viruses are mostly spread in regions with limited resources, where local laboratories are lacking the relevant equipment. Consequently, rapid diagnostic tests (RDTs) are critical for early detection of them. The EBOV glycoprotein (GP) mediates virus attachment and entry into host cells, while nucleoprotein is abundant in virus particles, both of which can induce strong immune responses and, therefore, are ideal antigens for vaccine and diagnostic studies. Many studies have identified the epitopes of these proteins, providing data for the development of Ebolavirus detection reagents [121,122,123]. Note that the glycoprotein (GP) 1,2 preproteins of EBOV and MBV species have shown that amino acid sequences of the N- and C-terminal regions share 31% homology. Hence, one report proposed that the three conserved B cell epitopes of (GP), validated by using in silico immunoinformatics technologies and in vitro enzyme immunoassays, may become the targets for RDTs of EBOV diseases and MBV diseases [123]. The innovation of this idea is to utilize the conservative epitopes of the homologous region to design a multifunctional RDT for screening all filamentous viruses (pan-filamentous viruses); after all, existing methods are specific for their own filoviruses.

#### 4.2.6. Hantaviruses 

Orthohantaviruses cause hemorrhagic fever with renal syndrome (HFRS) and orthohantavirus cardiopulmonary syndrome (HCPS). It is difficult to distinguish HFRS and HCPS from other tropical hemorrhagic diseases when relying solely on clinical manifestations, so serological assays are still necessary diagnostic tests. Whether HFRS or HCPS will appear after infection depends on the viral genotype and the reservoir species of different locations. The nucleocapsid proteins (NPs) have been found to be highly conserved among different hantavirus genotypes and are thus considered to be the ideal protein for diagnosing hantaviruses of different genotypes. In 2019, a publication reported that the Seoul orthohantavirus nucleoprotein (SHNP) sequence was used as a model to predict the linear B cell epitopes on SHNP and its immunogenicity was evaluated by in silico methods. In the end, two of nine potential immunogenic linear B cell epitopes—SHNP_(G72-D110)_ and SHNP_(P251-D264)_, which were highly conserved among species associated with HFRS—were found and validated in experiments. They are expected to be the diagnostic epitopes for HFRS-associated orthohantaviruses [124]. In addition, another team predicted the B cell epitopes of HFRS causing hantaviruses, and obtained a peptide with a 20 amino acid sequence length, which was suggested for use in geographic region-specific immunoassays [125]. Another research group intended to address the problem of detecting all genotypes causing HCPS and, therefore, predicted the first pan-specific epitope of NP IMASKSVGS/TAEEKLKKKSAF [126]. All of them predicted epitopes through computational methods, but the performance of diagnostic kits needs to be verified by extensive experiments. (Table 4).

### 4.3. Diagnosis in Parasitic Infections

Most parasitic diseases are diagnosed on the basis of finding parasite eggs or parasite bodies, but the serological test is still an important part of the diagnostic process for parasitic diseases. Some examples of epitopes being used in the diagnosis of parasitic diseases are described below. *Toxoplasma gondii* is capable of infecting an extremely wide range of mammals and birds, and is a major opportunistic pathogen with a high mortality rate in immunocompromised individuals. To date, the serological test remains the primary method to characterize *Toxoplasma gondii* identification, while most diagnostic kits use crude *Toxoplasma* antigens, with difficulties such as standardization and cost-effectiveness. A synthetic gene, named USM.TOXO1, encoding nine immunodominant epitopes of *T. gondii* antigens, was constructed by some researchers who cloned it into a pET32a expression vector. Then, a purified antigenic protein was obtained with 85.43% and 81.25% for diagnostic sensitivity and specificity, respectively, in IgG ELISA [128]. Furthermore, the epitopes of SAG1, SAG2A, and GRA1–GRA7, etc., were all studied and found to be possible in the development of diagnostic kits [129,130]. In addition, *Leishmania* is the causative agent of a complex neglected tropical disease—leishmaniasis—and is widespread in a large number of areas [131,132]. It is estimated that dozens of pathogens can cause human diseases that lead to different clinical symptoms in patients, ranging from cutaneous lesions to its fatal visceral form [131,133]. For the diagnosis of *Leishmania*, the most reliable and classical method relies on the microscopic visualization of the amastigote form of the parasite from tissue specimens after Giemsa staining, the sensitivity of which depends on the tissue [134]. The serological test is another important tool for the diagnosis of leishmaniasis infections, especially visceral leishmaniasis (VL). The recombinant protein K39 (rK39) is currently used in the diagnosis of VL, but its specificity needs to be further improved and it cannot be used to differentiate between infected and asymptomatic infected individuals. Many efforts have been invested in upgrading the diagnostic action of epitopes. The main direction is that a large number of epitopes located in strong antigenic proteins have been predicted, with the hope of improving the specificity of the method in human serum. A specific B cell epitope of hypothetical protein was predicted and evaluated in ELISA experiments to diagnose human VL, with 100% sensitivity and specificity [135]. Furthermore, one of the main strategies of VL control targets the canine reservoir, indicating that it is important to distinguish the serum of infected dogs from that of the uninfected. A bi-epitope linked by a Gly-Gly spacer, containing both an epitope corresponding to the N-terminal region of recLdVFA2 and another epitope, which were both obtained by mapping continuous B cell epitopes, was developed to diagnose canine VL in vitro. The sensitivity and specificity of this method were 98% and 99%, respectively [136,137,138]. Some epitope studies aim to determine the specific epitopes that can implement the observation of the prognosis of treatment or distinguish asymptomatic infection. Overall, the performance of epitopes in diagnosing *Leishmania* infection have a promising future. Schistosomiasis remains a serious public health problem, the causative agent of which is the genus *Schistosoma.* One of the essential means to effectively control and monitor schistosomiasis is to select diagnostic methods for early diagnosis [139,140]. Interestingly, a considerable number of selections of antigenic epitopes for schistosomes have been conducted by the peptide arrays technique to combine multiple effective peptides from different proteins. A recent manuscript evaluated the diagnostic performance of an immunodominant antigen from *S. japonicum*, *S. japonicum* saposin protein SjSAP4. Meanwhile, an epitope of the protein has also been identified. Although the sensitivity of the epitope is inferior to the antigen protein by comparison, the specificity of the epitope was found to be 100%, and the result of dual-epitope ELISA showed superior diagnostic performance than the single-epitope version. Considering the complex immunology and host–parasite interactions, the role of epitopes in the diagnosis of schistosomes can be made more significant with a rational choice of epitopes [141]. (Table 5).

### 4.4. Diagnosis in Fungal Infection

There are more than 6 million species of fungi inhabiting the earth, but only a few hundred can infect and cause disease in humans [159]. Fungi can not only cause infections from the most common superficial infection of skin and nails but also cause invasive fungal infections associated with high mortality rates [160], especially in some immunocompromised populations. Meanwhile, the infection rate of mycoses and resistance to antimycotic drugs appears to be increasing [161]. It is one of the greatest challenges to safe and early diagnosis of invasive fungal infections in routine clinical practice [160,161]. Because the available diagnostics for most fungal infections suffer from a lack of specificity, sensitivity, or both, including antigen detection test-an auxiliary tool for the diagnosis of invasive infections. Compared with the previous pathogens, the number of works that target diagnostic epitopes in fungi is substantially limited. To search for antigens with diagnostic potential, some mycological studies utilize the immunoproteomic approach to identify antigens on a large scale, followed by epitope prediction of antigens, including *Paracoccidioides* Spp. [162], *Histoplasma capsulatum* [163] and *Cryptococcus gattii* [164]. Although the diagnostic properties of epitopes need to be further validated, these studies provide new targets for the development of diagnostic reagents for fungi. 

In addition, some studies directly selected a single epitope in the antigen for testing, such as *Candida albicans*, which can cause systemic *C. albicans* infection. Heat shock protein 90 (Hsp90) and secreted aspartic protease (Sap) are two immunodominant antigens of *C. albicans*. Phage display technology is used to display epitopes of these proteins, but the study found that the diagnostic performance of a single epitope is not as good as its recombinant protein, which may be due to several epitopes in the recombinant protein. However, phage display technology is simpler and cheaper than traditional methods of chemical synthesis or separation and purification of antigens [165,166]. Similarly, two pieces of the literature have also reported the diagnostic ability of a single epitope of *Paracoccidioides brasiliensis* [167,168], one of which shows that the combined use of two synthetic peptides can reduce false negative results [169]. There are other studies that synthesize multi-epitopes into recombinant proteins for detection, including *C. albicans* [170], *Cryptococcus* spp. [171], and *Pneumocystis jirovecii* [172,173].

The culture examination of clinical specimens is usually used as the standard diagnosis of systemic mycosis, and the detection of fungal antigen or antibody is also one of the means for early diagnosis of invasive fungal infection, but for most fungal diseases, it is often unavailable [174]. All in all, the role of epitopes in the diagnosis of mycosis needs to be more explored to overcome the challenges that the wide antigenic variability of the clinical isolates the lack of standardization of serological.

## 5. Discussion and Conclusions

Serology remains an important diagnostic tool for infectious diseases, but an open question is a lack of sensitivity and specificity for the detection of certain diseases when using recombinant proteins as antigens. The existence of cross antigens is the main reason. As the smallest immune unit to stimulate the immune response, epitopes cannot be overestimated in the study of their structure and function. Given the specificity of epitopes, the search for epitope-based vaccine development, disease diagnosis and therapy has been ongoing for decades. Before, B cell epitopes were studied more compared with T cell epitopes for the serology tests, because the former are involved in humoral immunity. Moreover, although X-ray crystallography and NMR techniques, used in earlier years, are still reliable methods for determining epitopes at present, they are time-consuming, laborious, and costly [11]. Carefully, related methods for epitope mapping are evolving. Phage display technology and amino acid site-directed mutagenesis are two of them welcomed methods now, with the ability to high throughput screen of epitopes [48]. Even though they possess drawbacks as well, it is undeniable that these techniques have provided great help in immunology advances. Since the rapid development of bioinformatics technology and the advent of the genetic age, in silico methods have been increasingly applied in epitope prediction due to their numerous advantages such as convenience, rapidity, and low cost. Different predicting methods make use of different tools and software, which consist of many algorithms and parameters. Nonetheless, the existing prediction accuracy is still insufficient, especially for conformational B cell epitopes where there is insufficient information available for antigen–antibody structures and mimotopes, and no comprehensive and reliable dataset for training and testing. Different algorithms are based on different datasets, making it difficult to compare prediction performance. Moreover, based on statistical data, it is not yet possible to precisely distinguish epitopes from non-epitopes [24,175]. Accordingly, epitope prediction is expected to be measured by several computational methods to obtain a more exact result. There is still a long way to go to improve the accuracy of prediction results for LEs and CEs.

The work on applications of epitopes in the diagnosis has accelerated with the help of immunoinformatics technology. The use of epitopes in serological diagnostic tests aims to the improvement of detectability, sensitivity and specificity. Therefore, theoretically, many problems have the opportunity to be solved by the rational design of epitope combinations. The selection of multiple strongly immunogenic epitopes may enhance the sensitivity and specificity to distinguish between positive and negative sera [93]. In addition, it is possible to differentiate between different serotypes of pathogens by finding serotype-specific epitopes or to find pan-specific epitopes to detect all serotypes [114]. Even more, it gets the chance to differentiate between different stages of the disease by selecting chronophasic epitopes. For example, recovery from syphilis is traditionally dependent on the detection of nontreponemal rapid plasma reagin titers. However, it was proposed that titers are not as relevant to the prognosis of syphilis as might be expected [176]. Thus, epitopes in infection phase-dependent antigens are promising to evaluate the recovery of syphilis [177]. 

As described before, the application of epitopes in diagnosis has a few advantages, but a number of challenges remain. Firstly, the performance of existing B-cell epitope prediction methods is not satisfactory that may be related to the datasets, features and classifier, which indicates that methods need to be further improved to acquire a more accurate result [11]. Secondly, most of the epitopes used for diagnosis are represented by short peptides or recombinant proteins, making it difficult to simulate the natural conformation of the epitope, which may affect the diagnostic performance of the epitope. Last, there are relatively few studies that have actually been translated into clinical diagnostic kits. Although many studies have tested the performance of selected epitopes for the diagnosis of infectious diseases through certain experiments, some results may be influenced by certain factors, such as geographical location and ethnic background of the population being studied, thus, considerable efforts should be invested in obtaining adequate clinical trial data for the application of epitopes in clinical kits [57]. 

In conclusion, although research on antigenic determinants has obtained encouraging results, there are still many difficulties ahead of us. It is suggested that with the progress of technologies and the continuous efforts of scientists, epitopes will be more suitable to apply in the diagnosis of infectious diseases. 

## Figures and Tables

**Table 1:** Summary of prediction methods for B cell epitopes in this paper.

Methods		Year	Description	Availability
Linear Epitopes				
Propensity scales-based	Hopp and Woods [13]	1981	Hopp–Woods hydrophilicity.	Not stated
	PEOPLE [14]	1999	Antigenic index AG, which includes secondary structure, hydrophilicity, surface accessibility and flexibility.	Not stated
	BcePred [15]	2004	Combination of residue properties hydrophilicity, flexibility, polarity and surface solvent accessibility.	http://crdd.osdd.net/raghava/bcepred/index.html (accessed on 1 September 2022)
ML-based	BepiPred [17]	2006	Combine an HMM with an amino acid propensity scale.	Not currently available online
	ABCpred [18]	2006	Based on standard feed-forward (FNN) and recurrent neural network (RNN).	http://www.imtech.res.in/raghava/abcpred/ (accessed on 1 September 2022)
	SVMTriP [33]	2012	Use support vector machine (SVM) to combine the tripeptide similarity and propensity scores.	http://sysbio.unl.edu/SVMTriP/ (accessed on 1 September 2022)
	BepiPred 2.0 [19]	2017	Random forest (RF) algorithm trained on epitopes derived from antibody–antigen complex structures.	https://services.healthtech.dtu.dk/service.php?BepiPred-2.0 (accessed on 1 September 2022)
Conformational Epitopes				
Sequence-based	COBEpro [34]	2009	A two-step method, which first uses a SVM model to assign an epitopic propensity score to fragments within the given peptide sequence and then calculates an epitopic propensity score for each residue based on the scores in the first stage.	http://scratch.proteomics.ics.uci.edu (accessed on 1 September 2022)
	Bprediction [35]	2012	Adopt ensemble learning approach to incorporate various sequence-derived features, and develop an ensemble model.	Not currently available online
Structure-based	CEP [20]	2005	Use accessibility of residues and spatial distance cutoff to predict epitopes of protein antigens with known structures	Not currently available online
	DiscoTope [21]	2006	Use solvent accessibility, amino acid statistics and spatial information.	Not currently available online (DiscoTope2.0: https://services.healthtech.dtu.dk/service.php?DiscoTope-2.0) (accessed on 1 September 2022)
	ElliPro [22]	2008	Implement Thornton’s method, and together with a residue clustering algorithm, the MODELLER program and the Jmol viewer for predicting CEs.	http://tools.immuneepitope.org/ellipro/ (accessed on 1 September 2022)
	SEPPA [23]	2009	Employ the concept of “unit patch of residue triangle” to describe the local spatial context of protein surface and “clustering coefficient” to describe the spatial compactness of surface residues. Then, the two features are combined to predict epitopes.	Not currently available online (SEPPA3.0: http://www.badd-cao.net/seppa3/index.html) (accessed on 1 September 2022)
Mimotope-based	MIMOX [28]	2006	Map a single mimotope or a consensus sequence of a set of mimotopes onto the corresponding antigen structure and search for all of the clusters of residues that could represent the native epitope.	Not currently available online
	MimoPro [27]	2011	Operate a searching algorithm on a series of overlapping patches on the surface of a protein. These patches are then transformed to a number of graphs using an adaptable distance threshold regulated by an appropriate compactness factor.	Not currently available online
	PepMapper [24]	2012	An ensemble approach to incorporate two servers: Pep-3D-Search and MimoPro.	Not currently available online
Antibody-based	Shinji Soga [29]	2010	Predict epitopes for individual antibodies by narrowing down candidate epitope residues using the Propose ASEP index proposed in this method.	Not stated
	EpiPred [30]	2014	Combine conformational matching of the antibody–antigen structures and a specific antibody–antigen score.	http://www.stats.ox.ac.uk/research/proteins/resources (accessed on 1 September 2022)
	PEASE [36]	2014	Use antibody–antigen contact preferences, as well as other properties computed from the antibody sequence and antigen structure or sequence.	Not currently available online

**Table 2:** Brief introduction of epitope mapping technologies.

#	Epitope Mapping Technologies	Advantages	Disadvantages
1	X-ray crystallography	Gold standard method that provides detailed information about the epitope and paratope.	Laborious, time-consuming and complicated for eutectics.
2	NMR spectroscopy	Providing a dynamic picture of the antibody–antigen complex in solution.	Restricted to small proteins and peptides, time-consuming and complicated.
3	SPR	Highly sensitive, requires no additional biomarkers and can be dynamically tracked.	Low sensitivity for small molecule detection and high environmental requirements for the SPR sensor.
4	Pepscan	Low-cost and rapid.	Unable to provide complete epitope information.
5	Amino acid site-directed mutagenesis	Simple and quick to screen several hundreds or thousands of proteins.	Difficult to identify whether the mutation has disrupted the folding of the protein or is genuinely a key interacting residue.
6	Surface display technology	High-throughput screening, highly stable and easy to operate.	Different display systems have their own shortcomings; for example, the insertion of exogenous proteins in the phage display system may affect the assembly of phage, or the insertion of certain protein sequences into the bacterial surface system may lead to low protein secretion.

**Table 3:** Summary of diagnostic applications of bacterial epitopes in this paper.

Pathogens	Year	Associated Proteins/Peptides	Main Outcome			Ref.
			Sensitivity	Specificity	Others	
*Mycobacterium tuberculosis*	2007	Rv3872	P8 + P9: for pulmonary TB: 94%, for extra-pulmonary TB: 90%	Both 100%	/	[70]
	2013	ESAT-6 and CFP-10	/	/	E5 peptide and CFP10/ESAT-6 protein obtain similar results.	[56]
	2014	ESAT-6, CFP-10, CFP-21 and MPT-64	Combined peptides: for smear-positive pulmonary TB: 83.3%, for smear-negative pulmonary TB: 62.5%, for sarcoidosis: 4.16%	100%	/	[57]
	2022	Rv1981c, Rv2659c, Rv3879c	/	/	The polypeptide molecule can induce robust immune responses, and may be a new diagnostic biomarker for latent TB infection	[58]
*Staphylococcus aureus*	1998	SEA and SEB	/	/	The mAbs for epitopes were able to quantitate the native SEA or SEB at nanogram levels.	[62]
	2015	PSau5 and PSau7	/	/	A competitive ELISA based on epitopes can detect *S. aureus* down to 10^4^ CFU mL^−1^	[63]
*Leptospira*	2006	Phage mimotopes	/	/	Mimotopes reacting with both mAbs and patients’ sera have potential for further use as diagnostic reagent.	[71]
	2008	OmpL1, LipL21, and LipL32	/	/	The multiepitope protein recognized IgG and IgM in all the sera that were MAT positive.	[72]
	2010	Hap1/LipL32	/	IgM: 89% IgG: 100%	The peptide is an earlier serological diagnosis of human leptospirosis than MAT.	[69]
	2014	LigA	Epitopes 1 and 2: 97.9%	Epitopes 1 and 2: 99.1%	/	[65]
	2016	LipL21(r-I-LipL21)	92.59%	92.86%	/	[66]
	2017	LK90543 and LK901110 of LigA	77~89%	93~96%	The results of mAb-based dot blot ELISA; The mAbs are targeted for epitopes.	[67]
*Chlamydia trachomatis*	2008	OmcB	23.9%	94.3%	/	[73]
	2018	11 peptides from 8 proteins	11 peptides: 91.8% 5 optimal peptides: 86.5%	Both 98%	/	[74]
	2018	12 peptides from different proteins	Ctr Mix1:94%	Ctr Mix1: 98%	/	[75]
*Chlamydia pneumoniae*	2019	Mixed peptides from different proteins	CpnMixF12: 91%	CpnMixF12: 95%	/	[76]
*Borrelia burgdorferi*	2014	OppA2	OppA (191-225): 44.2%	OppA (191-225): 95.5%	/	[77]
	2016	OspC	/	/	Six OspC epitopes capable of distinguishing between Lyme disease patient and healthy control sera.	[78]
	2017	OspA and OspC	A/C-2 and A/C-7.1: 80.17% and 91.37%	A/C-2 and A/C-7.1: 52.83% and 73.58%	/	[79]
	2019	BBK32	BBK32(51–80): 33.3%	BBK32(51–80): 94.7%	/	[80]
	2021	Epitope motifs	77%	99%	Results of diagnosing early Lyme disease	[81]
*Borrelia miyamotoi*	2020	Several peptides identified by peptide array	/	/	The panel of linear peptides may have greater potential for differential diagnosis.	[82]
*brucella*	2016	OMP16, 2b, 31, and BP26 (periplasmic protein)	88.89%	85.54%	/	[83]
	2021	OMPs	96.49% (95% CI, 87.89 to 99.57)	94.44% (95% CI, 81.34 to 99.32	The results are obtained at optimum cut off value 0.6195	[84]
	2021	Recombinant protein (OMP 22, 25, and 31)	84.37%	83.78%	/	[85]
	2021	Multiepitope protein (BP26, OMP31, 16, 2b and 25)	92.38%	98.35%	PPV: 98.26% NPV:91.67%.	[86]

**Table 4:** Summary of diagnostic applications of virus epitopes in this paper.

Pathogens	Year	Associated Proteins/Peptides	Main Outcome			Ref.
			Sensitivity	Specificity	Others	
SARS-CoV-2	2020	S protein (S14P5 and S21P2)	/	/	Two epitopes are strongly recognized by sera from COVID-19 convalescent patients. One is specific to SARS-CoV-2, the other region, which could potentially function as a pan-SARS target.	[88]
	2020	S14P5, S20P2, S21P2 and N4P5	N4P5: >96%	N4P5: 100%	The magnitude of IgG responses to S14P5, S21P2 and N4P5 were strongly associated with disease severity	[96]
	2020	orf1a/b, S, and N proteins	/	/	Positive rate IgG:71.4% IgM: 57.2%	[90]
	2020	27 proteins	/	/	Nucleocapsid protein and a highly antigenic GGDGKMKD epitope were identified as ideal antigens to be used in the development of serodiagnostic assays.	[94]
	2021	S protein	/	/	S14P5 + S21P2 + P104: Positive reaction rate for all patients and asymptomatic infections: 92.7% and 86.7%	[89]
	2021	N and S protein	/	/	Several B-cell epitopes having potential diagnostic performance have been identified	[91]
	2021	S region	/	/	Selected four peptides for SARS-CoV-2 diagnosis in silico.	[92]
	2021	ORF8 protein	/	/	Peptides 1, 2, 8 and 15 were recognized in ≥75% COVID-19 patients.	[97]
Epstein–Barr virus	2006	Peptides F1, A3, gp125, and A2	F1: 88%, A3:85% gp125: 71% A2: 54% gp125 + F1: 99%	100%	/	[101]
	2011	LMP2	/	/	Positive rates in the NPC group: Epitope1/2/3: 90.82% / 62.56% / 69.39%	[103]
	2016	LMP2	52.84%	95.40%	/	[102]
	2018	LMP2	91.91%	93.14%	/	[98]
Dengue virus	2001	NS1 of DENV-1	95%	100%	/	[112]
	2003	NS1 of DENV-2	/	/	This mAb and its epitope-based peptide antigen will be useful for serologic diagnosis of DENV-2 infection.	[113]
	2013	E protein of DENV 1 to 4 serotypes	71.7%	100%	/	[106]
	2018	E protein of DENV-1	E1: 82.5% E7: 79.2% E1 + E7: 85.0%	E1: 94.6% E7: 92.9% E1+ E7:96.4%	/	[107]
	2019	NS4B protein	IgG: 87.88% IgM: 79.17%	IgG: 93.55% IgM: 82.61%	/	[114]
	2019	E protein	E01: 100%	E01: 75%	/	[109]
	2020	E and NS1proteins	73.33–96.66%	82.14–100%	/	[108]
	2020	E protein	/	/	Substituting key residues for alanine within linear epitopes on the surface of the DENV E protein abolishes the contribution of some cross-reacting epitopes to its antigenicity.	[111]
Hepatitis virus	2013	HBcAg recombinant hepatitis B core multiepitope antigen	/	/	Performance of recombinant antigen responds as well as the commercial antigen.	[115]
	2021	N-terminal residues of HBeAg	/	100%	A novel HBeAg immunoassay using high-affinity mAbs recognizing unique N-terminal epitope on precore protein, eliminating the confounding signal from the secreted HBcAg.	[116]
	2006	Recombinant multiepitope protein (core 1b, core 3g, NS3, NS4 I, NS 4 II, and NS5.)	99.8%	100%	Sensitivity and specificity can be comparable with commercially available anti-HCV EIA	[117]
	2009	Core protein	/	/	40.7% patients with occult HCV infection showed IgG anti-HCV core reactivity.	[120]
	2011	Core, NS3, NS4 and NS5 proteins	/	/	With good sensitivity and specificity	[118]
	2013	Epitope arrays	/	/	Combinatorial epitopes proved to be effective for the discrimination between positive and negative sera as well as serotyping of HCV.	[127]
	2018	E2 region	/	/	HC-13 has a high degree Of specificity in E2 region among the genotypes.	[119]
Ebolavirus	2013	NP	/	/	/	[121]
	2014	GP, NP, and VP40 and VP35	/	/	The B-cell epitopes identified may represent important tools for developing new antibody-based detection methods.	[122]
	2018	GP	/	/	These conserved B cell epitopes of GP1, 2 and their derivative antibodies are targets presently for development of RDTs for Ebolavirus disease.	[123]
Hantaviruses	2017	NP	/	/	The reported pan-specific epitopes can be developed for test detecting antibodies to hantaviruses causing HCPS	[126]
	2017	NP	/	/	It predicted a conserved 20-mer peptide used for development of geographic region-specific immunoassays	[125]
	2019	NP	/	/	SHNP_(G72-D110)_ and SHNP_(P251-D264)_ epitopes are promising targets to development of highly specific tools to HFRS orthohantavirus diagnosis.	[124]

**Table 5:** Summary of diagnostic applications of parasitic epitopes in this paper.

Pathogens	Year	Associated Proteins/Peptides	Main Outcome			Ref.
			Sensitivity	Specificity	Others	
*Leishmania*	2015	Multiepitope proteins PQ10 and PQ20	PQ10:88.8% PQ20:84.9%	PQ-10: 80% PQ-20: 65%	PQ10 was able to detect 80% of asymptomatic infected dogs.	[137]
	2022	Chimeric protein ChimLeish (LiHyT, LiHyD, LiHyV, and LiHyP)	100%	100%	For tegumentary leishmaniasis diagnosis.	[142]
*Leishmania donovani*	2017	P_1_P_2_ (P_1_:RFFVQGDGIGQHSLQEALERR and P_2_:RRVAVLVLLDRL)	P_1_P_2_: 90% ICT strip: 100%	P_1_P_2_:100% ICT strip: 95.2%	Colloidal gold conjugated anti-P1P2 antibody ICT strip anti-P1P2 antibody	[143]
	2017	recLdVFA2	98%	99%	For canine VL	[136]
*Leishmania infantum*	1998	LiP2a, LiP2b, LiP0 and H2A	79–93%	96–100%	For canine VL	[144]
	2005	K9, K26, and K39	Human/canine: 82%/96%	Both 99%	/	[145]
	2011	Peptides: PSLc1- PSLc10	PSLc10: 88.70%	PSLc8 and Mix10: 95.00%	Best results of peptides in serum samples from symptomatic and asymptomatic dogs	[146]
	2017	B10 Peptide/Phage and C01Peptide/Phage	90.5%/100% and 91.5%/92.3%	89.9%/98.1% and 85.5%/98.1%	/	[147]
	2018	6 peptides of 3 proteins (Protein ID: LinJ.30.2730, LinJ.32.0280, LinJ.27.0980)	Peptide-6, Mix I, II, III and IV: 100.00% (CI 95%: 94.87–100.00%)	Mix IV: 100.00%, (CI 95%: 97.36–100.00), Peptide-6 and Mix III: 99.28%, (CI 95%: 96.03–99.98)	Mix IV have the ability to identify VL cases and simultaneously to discriminate infections caused by *Trypanosoma cruzi* parasite with high accuracy (100.00%)	[148]
	2019	Peptide EpQ11	79–84%	/	None of the sera from nonendemic healthy control patients were positive.	[149]
	2020	Synthetic peptide PeptC of protein LiHyC	100%	100%	/	[135]
	2021	Peptides: Pep2, Pep3 and Pep4	100%	100%	Have potential to diagnose VL and VL/HIV coinfection.	[150]
*Toxoplasma gondii*	2012	GRA1, GRA2, GRA4, SAG1, NTPase1, NTPase2 and MIC3	69%	84%	Overall sensitivity is 69%. The assay has different diagnostic sensitivity in different types of patients, as can be seen in the references	[151]
	2012	SAG1, SAG2, SAG3, GRA5, GRA6, and P35	IgG: 94.4% IgM: 96.9%	IgG and IgM: 100%	/	[152]
	2017	SAG1, GRA2 and GRA7	85.43%	81.25%	/	[128]
	2021	SAG1, GRA1, ROP2, GRA4, and MIC3	79.1%	88.6%	IgG ELISA	[132]
	2021	BCLA	/	/	Peptides significantly increased the test sensitivity.	[153]
*Schistosoma mansoni*	2016	FLDNF	/	/	/	[154]
	2017	7 proteins	96.15%	100%	The ELISA performance is achieved by using peptide 5, which could discriminate between individuals living in an endemic area that were actively infected from those that were not.	[155]
	2021	SmSPI	/	/	Three predicted immunoreactive epitopes of SmSPI are potential biomarkers for serodiagnostic Schistosomiasis.	[156]
*Schistosoma japonicum*	2019	SjSP-13	76.7% (95% CI: 68.8–84.5%)	100%	Two adjacent peptides (p7 and p8)	[157]
	2020	SjEV proteins	/	/	Combined epitope protein demonstrated a modest sensitivity for detection of *S. japonica*	[140]
	2022	SjSAP4 and SjSP-13	84.0%	100%.	A dual epitope-ELISA (SjSAP4-Peptide + SjSP-13V2-Peptide-ELISA)	[141]
*Plasmodium* *falciparum*	2016	M.RCAg-1 (11 epitopes)	/	/	M.RCAg-1 was well-recognized by the naturally acquired anti-malaria antibodies and can be used as a tool for assessing malaria transmission intensity in the border area of China-Myanmar.	[158]

## Data Availability

Not applicable.

## Abbreviations

PCR	Polymerase chain reaction	COVID-19	Coronavirus disease 2019
LEs/CEs	Linear/conformational epitopes	S	Spike
B/TCRs	B/T cell receptors	E	Envelope
MHC	Major histocompatibility complex	M	Membrane
Igs	immunoglobulins	N	Nucleocapsid
ML	Machine learning	ORF	Open reading frame
HMM	Hidden Markov model	EBV	Epstein–Barr virus
ROC	Receiver operating characteristic	NP	Nucleoprotein
ANN	Artificial neural network	NPC	Nasopharyngeal carcinoma
AUC	Area under the curve	LMP2	Latent membrane protein 2
ASEP	Antibody-specific epitope propensity	DENV	Dengue virus
FNN	Feed-forward neural network	NS1	Non-structural protein 1
RNN	Recurrent neural network	HBV/HCV	Hepatitis B/C virus
SVM	Support vector machine	HBeAg/HBcAg	Hepatitis B e/core antigen
RF	Random Forest	EBOV	Ebolavirus
NMR	Nuclear magnetic resonance	MBV	Marburgvirus
SPR	Surface plasmon resonance	RDTs	Rapid diagnostic tests
Pepscan	Peptide scanning	GP	Glycoprotein
ELISA	Enzyme-linked immunosorbent assay	HFRS	Hemorrhagic fever renal syndrome
WB	Western blotting	HCPS	Hantavirus cardiopulmonary syndrome
SARS-CoV-2	Severe acute respiratory syndrome coronavirus 2	SHNP	Seoul orthohantavirus nucleoprotein
TB	Tuberculosis	VL	Visceral leishmaniasis
RD	Regions of difference	VP	Viral protein
CFP-10	Culture filtrate protein	SjSAP4	*S. japonicum* saposin protein
ESAT-6	Early secretory antigenic target	SjSP-13	*S. japonicum* saposin-like protein
OMPs	outer membrane proteins	SmSPI	*S. mansoni* Serine protease inhibitor
MAT	microscopic agglutination test	BCLA	Brain Cyst Load-associated Antigen
SEA/SEB	Staphylococcal enterotoxin A/B	Hsp90	Heat shock protein 90
Opp	Oligopeptide permease	Sap:	Secreted aspartic protease
Osp	Outer surface protein	SjEV	*S. japonicum* Extracellular vesicles
PPV/NPV	Positive/negative predict value	ICT	Immunochromatographic test
